# Carbapenem Resistant *Aeromonas hydrophila* Carrying *bla*_*cphA*7_ Isolated From Two Solid Organ Transplant Patients

**DOI:** 10.3389/fcimb.2020.563482

**Published:** 2020-10-26

**Authors:** Evann E. Hilt, Sean Patrick Fitzwater, Kevin Ward, Annabelle de St. Maurice, Sukantha Chandrasekaran, Omai B. Garner, Shangxin Yang

**Affiliations:** ^1^Department of Pathology and Laboratory Medicine, University of California, Los Angeles, Los Angeles, CA, United States; ^2^Department of Pediatrics, University of California, Los Angeles, Los Angeles, CA, United States

**Keywords:** carbapenem resistant, *Aeromonas hydrophila*, CphA7, carbapenemase, metallo- beta-lactamase

## Abstract

*Aeromonas hydrophila* resides in a variety of aquatic environments. Infections with *A. hydrophila* mainly occur after contact with fresh or brackish water. Nosocomial infections with *A. hydrophila* can also occur. *A. hydrophila* infections can be difficult to treat due to both intrinsic and acquired antimicrobial resistance (AMR) mechanisms. In 2018–19, we isolated multi-drug resistant (MDR) *A. hyrodphila* from two solid organ transplant patients with intra-abdominal infections. We aimed to characterize their AMR mechanisms and to determine their genetic relatedness to aid epidemiological investigation. We performed whole genome sequencing (WGS) using Illumina MiSeq and Nanopore MinIon on 3 *A. hydrophila* isolates, with one isolate from Patient A (blood) and two isolates from Patient B (abdominal and T-tube fluid, isolated 2 weeks apart). Phenotypic assays included: Broth Microdilution (BMD), Modified Hodge Test (MHT), Modified Carbapenem Inactivation Method (mCIM), and EDTA Carbapenem Inactivation Method (eCIM). Data analyses were performed using CLCbio and Geneious. AMR genomic analysis revealed that all three isolates possess chromosomally encoded genes including *bla*_*OXA*−12_(oxacillinase)*, bla*_*cepS*_(AmpC), and *bla*_*cphA*7_(metallo-beta-lactamase). All isolates tested strongly positive by MHT and mCIM, but only Patient B's second isolate (after 2 weeks of meropenem treatment) tested positive by eCIM. More intriguingly, Patient B's first isolate (before meropenem treatment) tested falsely susceptible to carbapenems by BMD, suggesting *bla*_*cphA*7_ gene was not expressed constitutively. Phylogenetic analysis showed the two isolates from Patient B were highly similar with only 1 SNP difference. The isolate from Patient A only differed from Patient B's isolates by 35 and 36 SNPs, respectively, suggesting close genetic relatedness. Further epidemiological investigation is undergoing. We report the first cases of CphA-mediated carbapenem resistant *A. hydrophila* in the U.S. It is concerning that 1 out of 3 isolates tested falsely susceptible to carbapenems by BMD despite clear carbapenemase production shown by strongly positive MHT and mCIM. In both cases, meropenem was initially used to treat the patients. Clinicians and microbiologists in the US should be aware of the emerging MDR *Aeromonas* nosocomial infections and the potential false carbapenem susceptible results due to CphA-type carbapenemase, which may be induced during treatment.

## Introduction

*Aeromonas hydrophila* is a Gram-negative bacillus that resides in a variety of aquatic environments (Hazen and Fliermans, 1979). Infections with *A. hydrophila* mainly occur after contact with fresh or brackish water. These infections can range from mild illness such as cellulitis or gastrointestinal disease, to serious disease such as sepsis and necrotizing fasciitis (Lee et al., 2008; Wu et al., 2009; Janda and Abbott, 2010). Nosocomial infections with *A. hydrophila* can occur and in some cases these infections are associated with contaminated medical devices such as catheters used in hemodialysis treatment (Lin et al., 1996; Khalil et al., 2013).

*A. hydrophila* infections can be difficult to treat due to both intrinsic and acquired antimicrobial resistance (AMR) mechanisms. The main mechanism of intrinsic resistance is chromosomally encoded ß-lactamases including Ambler class C cephalosporinases, class D penicillinases and class B metallo-ß-lactamases (MBLs) (Janda and Abbott, 2010). The most common MBL found in *A. hydrophila* is CphA that has a very specific substrate profile: highly active on carbapenems but not penicillin and cephalosporins (Segatore et al., 1993; Wu et al., 2012). CphA is also found in other clinically relevant *Aeromonas* species: *A. bestiarum, A. caviae, A. sobria, A. veronii*, and *A. jandaei* (Rossolini et al., 1995). Disseminated infections by CphA carrying *A. hydrophila*, primarily bacteremia, have mainly been reported in Asian and South American countries including Taiwan (Wu et al., 2007, 2011, 2012), Australia (Sinclair et al., 2016), and Colombia (Rosso et al., 2019).

CphA carrying *A. hydrophila* are resistant to extended-spectrum cephalosporins but are susceptible to monobactams such as aztreonam (Janda and Abbott, 2010). Reports have shown that carbapenemase-mediated resistance due to CphA is not easily detected by common *in vitro* susceptibility methods (Rossolini et al., 1995). Susceptibility tests with a large inoculum such as the Modified Hodge Test (MHT) have been shown to accurately detect carbapenem resistance and carbapnemase activity in CphA carrying strains of *Aeromonas* (Wu et al., 2011, 2012).

In 2018–19, we isolated several multi-drug resistant (MDR) *A. hydrophila* isolates from two solid organ transplant patients both presenting with intra-abdominal infections and subsequent bacteremia. We aimed to characterize their AMR mechanisms and to determine their genetic relatedness to aid epidemiological investigation.

## Materials and Methods

### Bacterial Isolates, Antimicrobial Susceptibility, and Phenotypic Testing

A total of three *Aeromonas* isolates were included in this study. The first (A-1) was from a patient's blood culture (Patient A), while the other two were isolated 2 weeks apart from the intra-abdominal abscess (B-1) and T-tube fluid (B-2) from Patient B. These three isolates were identified as *Aeromonas hydrophila* from the original culture plates using Matrix Assisted Laser Desorption Ionization-Time of Flight mass spectrometry (Biomerieux). The antimicrobial susceptibility testing was performed using Broth Microdilution (BMD) following guidelines set forth by the Clinical and Laboratory Standards Institute (CLSI) (CLSI, 2016). The Modified Carbapenem Inactivation Method (mCIM) and the EDTA-Carbapenem Inactivation Method (eCIM) were performed following the CLSI of the M100 guidelines (29th edition) (Clinical Laboratory Standards Institute, 2019). Modified Hodge Test (MHT) was also performed (Amjad et al., 2011). A positive control (MHT positive Klebsiella pneumoniae ATCC1705) and a negative control (MHT negative Klebsiella pneumoniae ATCC1706) were included in all the three phenotypic carbapenemase tests.

### Whole Genome Sequencing (WGS)

The three *Aeromonas* isolates were sequenced with both short-read and long-read sequencing technologies. In brief, for short-read sequencing, extraction of the DNA from the isolates was done using the QIAGEN EZ1 DNA Tissue Kit (Germantown, MD) and the EZ1 Advanced XL (Germantown, MD). The libraries were prepared for sequencing using the Nextera DNA Flex Library Prep (Illumina; San Diego, CA). These prepared libraries were then loaded onto the Illumina MiSeq (San Diego, CA) with the 2 × 250 pair-read protocol. A positive control using *Escherichia coli* ATCC® 25922 DNA and a negative control of just water were run alongside the three *Aeromonas* isolates. For long-read sequencing, genomic DNA was extracted using Qiagen AllPrep Mini Kits (Germantown, MD) on the QIAcube automated system (Germantown, MD). Library preparation for sequencing on the Oxford Nanopore Technologies system was performed using the Native Barcoding Kit 1D and Ligation Sequencing kits, and run using FLO-MIN107 flow cells on the MinION system, following the manufacturer's instructions (Wick et al., 2017a). Base calling for Nanopore reads were completed using Guppy (v2.1.3, Oxford nanopore Technologies), and sequence reads were demultiplexed using Porechop (v0.2.4, https://github.com/rrwick/Porechop). Hybrid genome assembly was completed using Unicycler (v0.4.8-beta) (Wick et al., 2017b). Genomes were submitted to NCBI and can be found under BioProject PRJNA648413.

### Bioinformatics

Mapping of the three *Aeromonas* isolates to reference strains were performed using both CLCbio Workbench Version 12 (Qiagen, Germany) and Geneious Prime 2.1 (Biomatters, New Zealand). The Kmer-based phylogenetic tree and single nucleotide polymorphism (SNP)-based analyses were done using CLCbio Workbench Version 12 (Qiagen, Germany). Specifically, the Kmer-based phylogenetic tree was performed using Feature Frequency Profile which is an alignment free genome comparison (Sims et al., 2009). The AMR prediction analyses were performed using Nucleotide Database (DB) with QIAGEN Microbial Insight—Antimicrobial Resistance (QMI-AR) as the reference database and the parameters of a minimum percent identity and percent length of 90% on CLCbio Workbench Version 12 (Qiagen, Germany). Center of Genomic Epidemiology tools, including KmerFinder (for closely related strain) (Hasman et al., 2014; Larsen et al., 2014) and PlasmidFinder (for plasmid types) (Carattoli et al., 2014) were also used. Plasmids were annotated using RAST (Aziz et al., 2008).

## Results

### Clinical History

Patient A had a history of megacystitis, hypoperistalsis syndrome, renal failure requiring hemodialysis, multiple drug allergies, and a history of small bowel transplant complicated by rejection and subsequent ex-plantation. The patient developed end-stage liver disease due to chronic exposure to total parental nutrition. Patient A was initially admitted to the hospital for a foot infection and received treatment with broad spectrum antibiotics including meropenem. During to hospitalization, the patient had a wound culture from the site of a previous catheter insertion that grew multidrug resistant *Aeromonas* classified as *Aeromonas hydrophila group*, however this isolate was not able to be sequenced. Two weeks later the patient had a fever and a blood culture was obtained which grew multidrug resistant *Aeromonas* (A-1). Patient A was treated with broad spectrum antibiotics initially and ultimately completed a course of amikacin. The patient's hospital course was complicated by a gastrointestinal bleed, bacteremia, and fungemia. Patient A died several months later after transitioning to comfort care.

Patient B had a history of hypertension, polycystic liver-kidney disease who was initially admitted to our medical center for liver transplant. The patient's post-transplant course was complicated by bacteremia, cardiac arrest, and cerebral infarcts. Patient B received multiple courses of broad-spectrum antibiotics including meropenem during hospitalization. On 9/23/19, the patient had a surgical drain with milky drainage which grew multidrug resistant *Aeromonas* (B-1). Again, on 10/10/19 there was further drainage from a surgical drain which grew multidrug resistant *Aeromonas* (B-2). Patient B was treated with ceftazidime/avibactam and polymyxin/colistin. Patient B died 2 months later after transitioning to comfort care.

The Infection Prevention team conducted a thorough investigation to determine whether there was a correlation between Patient A and B's infection. The patients were admitted to the hospital during different time periods separated by over 6 months. No procedural items (e.g., duodenoscope) were shared between the two patients. Cleaning protocols for the rooms and point of care filter replacements for faucets were reviewed. Surveillance culture results of the dialysis machines were reviewed and had no evidence of contamination.

#### Phylogenetic Analysis

**Figure 1:**
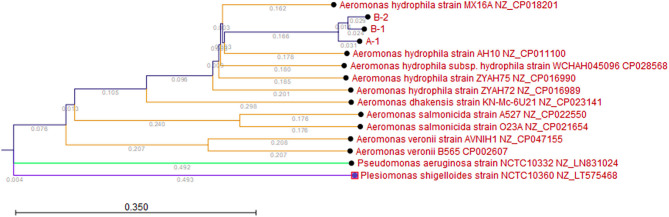
Kmer Tree. Kmer analysis was performed with the three *A. hydrophila* isolates (A-1, B-1, and B-2) and five reference *A. hydrophila* (Genbank IDs: NZ_CP018201, NZ_CP011100, NZ_CP016990, NZ_CP028568, NZ_CP016989), two reference *Aeromonas salmonicida* (Genbank IDs: NZ_CP022550, NZ_CP021654), two reference *Aeromonas veronii* (Genbank IDs: NZ_CP046155, NZ_CP002607), one reference *Aeromonas dhakensis* (Genbank ID: NX_CP023141), one reference *Pseudomonas aeruginosa* (Genbank ID: NZ_LN831024), and one *Plesiomonas shigelloides* (Genbank ID: NZ_LT575468). Branch lengths are shown to show the relatedness.

**Figure 2:**
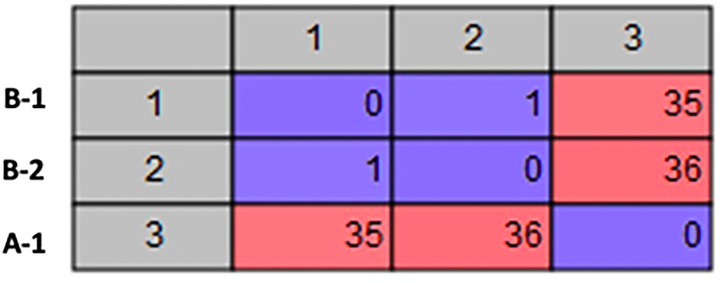
SNP Analysis. Matrix showing the number of SNP differences between each of the *A. hydrophila* isolates.

The Kmer Tree analysis revealed that the three *Aeromonas* isolates are all closely related to an *Aeromonas hydrophila* MX16A strain (NZ_CP018201) isolated from a water source in China in 2012 (Figure 1). Mapping of the three isolates to this reference strain showed an average Pairwise identity of 98.6% with a coverage of 94.4% (Table 1). We performed a SNP analysis and found that the two isolates from Patient B were highly similar with only 1 SNP difference detected (Figure 2). This SNP difference was in the gene that encodes for the protein RodA and appeared to cause a neutral amino acid change (Supplementary Table 1). RodA is a peptidoglycan glycosyltransferase important for cell wall elongation (Henriques et al., 1998). The isolate (A-1) from Patient A differed from B-1 and B-2 by 35 and 36 SNPs, respectively, suggesting a close genetic relatedness among these bacteria. A complete list of the SNPs among the 3 isolates are shown in Supplementary Table 1.

**Table 1:** Mapping statistics of the three *A. hydrophila* Isolates to NZ_CP018201.

**Isolate**	**Pairwise identity**	**Mean coverage**	**Ref-seq percentage**
2018–1	98.5%	63.4	94.0%
2019–1	98.6%	48.8	94.5%
2019–2	98.6%	54.3	94.6%
Mean	98.6%	55.5	94.4%

#### AMR Genotypic Analysis

AMR genotypic analysis revealed that all three isolates possess three different chromosomally encoded ß-lactamases: *bla*_*OXA*−12_(oxacillinase)*, bla*_*cepS*_(AmpC), and *bla*_*cphA*7_(metallo-beta-lactamase) (Table 2). The two isolates from the Patient B (B-1 and B-2) had additional AMR genes detected conferring resistance to the following antibiotic classes: aminoglycosides, fluoroquinolones, phenicols, macrolides, and trimethoprim-sulfonamide (Table 2). Hybrid sequencing revealed that these additional AMR genes were on plasmids (Table 3). We identified a novel plasmid (Plasmid 1) present in both isolates from Patient B that has 123,554 bp and carries 185 genes (Supplementary Tables 2, 3). BLAST analysis showed only 20% of the sequences in this plasmid matched to any known plasmids (Table 3). These plasmids have many hypothetical proteins based on RAST data (Supplementary Tables 2, 3); AMR gene analysis of this plasmids identified genes conferring to resistance to beta-lactams (*bla*_*SHV*−5_), aminoglycosides (*aadA2* and *aph(3*′*)-la*), fluoroquinolones (*qacH*), macrolides (*mphA*) and trimethoprim-sulfonamide (*dfrA12* and *sul1*) (Tables 2, 3).

**Table 2:** Antimicrobial genotypic prediction of the three *Aeromonas hydrophila* isolates.

**Antibiotic class**	**Antibiotic gene**	**A-1**	**B-1**		**B-2**		
		**Chromosome**	**Chromosome**	**Plasmid_1**	**Chromosome**	**Plasmid_1**	**Plasmid_2**
Beta-Lactams	bla_OXA−12_	✓	✓		✓		
	bla_cepS_	✓	✓		✓		
	bla_cphA7_	✓	✓		✓		
	bla_OXA−10_						✓
	bla_SHV−5_			✓		✓	✓
Aminoglycoside	aadA2			✓		✓	
	aph(3′)-la			✓		✓	
	ant(3″)-la						✓
	aac(6′)-lld						✓
	aadA13						✓
Trimethoprim-Sulfonamide	dfrA12			✓		✓	
	sul1			✓		✓	✓
	sul2						✓
Fluoroquinolone	qacH			✓		✓	✓
Phenicol	catB3						✓
	floR						✓
Macrolide	mphA			✓		✓	

**Table 3:** Hybrid sequence plasmid results.

**Isolate**	**Plasmid number**	**Length (base pairs)**	Genes^*^	**BLAST results**			
				**Plasmid description**	**Query**	**Percent identity**	**Accession**
B-1	Plasmid 1	123,554	184	*K. pneumoniae* AR_0120 plasmid tig00000500 pilon	19%	99.55%	CP021834.1
B-2	Plasmid 1	123,554	185	*K. pneumoniae* AR_0120 plasmid tig00000500 pilon	19%	99.56%	CP021834.1
	Plasmid 2	129,067	183	*K. pneumoniae* plasmid pHM881QN	94%	99.98%	LC055503.1

* *Based on RAST annotation results*.

The second isolate from the Patient B (B-2) appeared to have additional AMR genes detected compared to the first isolate (B-1) (Table 2). PlasmidFinder revealed an IncC-type plasmid present in isolate B-2 but not in the other two isolates. Hybrid assembly of both Illumina and NanoPore sequencing data confirmed that B-2 has a 129,067 bp plasmid (Plasmid 2) which carries 183 genes (Supplementary Table 4). BLAST analysis showed that this second plasmid in B-2 is 94% similar to a plasmid found in *Klebsiella pneumoniae* known as pHM881QN. It is a IncA/C plasmid isolated in Japan (GenBank ID: LC055503.1). The additional AMR genes on this plasmid in B-2 include the ß-lactamase *bla*_*OXO*−10_, aminoglycoside resistance genes *ant(3*″*)-la, aac(6*′*)-lld* and *aadA13*, phenicol resistance genes *catB3* and *floR*, and trimethoprim/sulfonamide resistance gene *sul2* (Tables 2, 3). The additional plasmid found in B-2 suggested that either this isolate gained a plasmid or that the antibiotic treatment of the patient with meropenem had selected this sub-population from a mixed bacteria population. Further analysis is undergoing to polish and finalize the complete genomes of these two plasmids.

#### Correlation Between AMR Genotypes and Phenotypic Susceptibility and Carbapenemase Profiles

**Figure 3:**
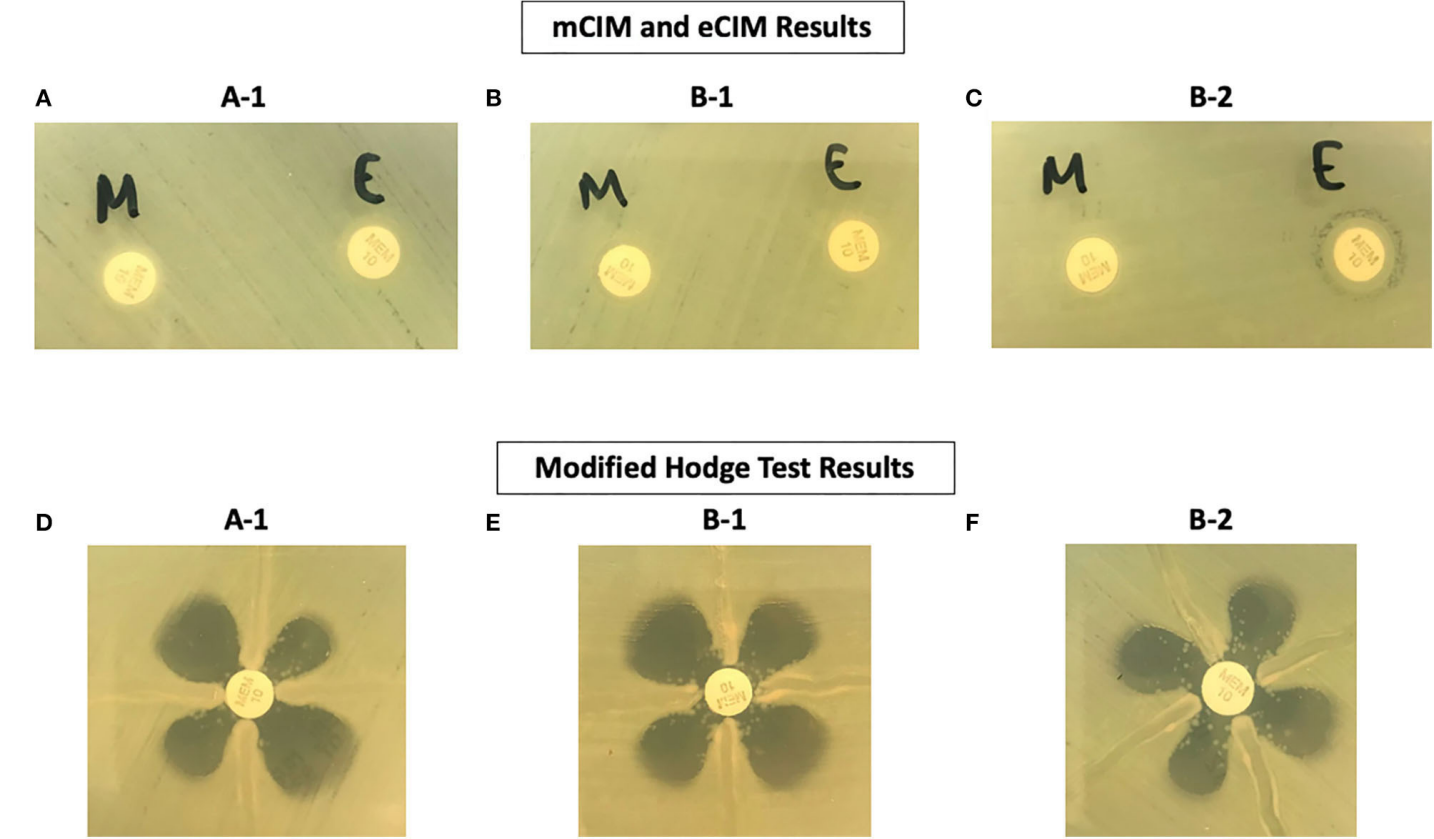
Phenotypic Test Results of Three *A. hydrophila* Isolates. **(A–C)** Are results from the mCIM and eCIM tests. **(D–F)** Are results from the MHT.

The phenotypic antimicrobial susceptibility results of the three *A. hydrophila* isolates demonstrated several interesting patterns (Table 4): (1) all isolates are resistant to cefazolin, piperacillin/tazobactam, and all 3rd generation cephalosporins, which is consistent with the presence of the oxacillinase gene *bla*_*OXA*−12_ and the AmpC gene *bla*_*cepS*_; (2) Isolate B-1 had elevated cefepime MIC due to the additional ESBL gene *bla*_*SHV*−5_ (Gutmann et al., 1989); (3) Isolate B-2 (after meropenem treatment) exhibits full resistance to all beta-lactams except ceftazidime/avibactam due to the additional beta-lactamase genes *bla*_*SHV*−5_ and *bla*_*OXA*−10_(Gutmann et al., 1989), as well as the apparently induced MBL gene *bla*_*cphA*7_ expression, which is shown by the only positive eCIM result. Interestingly, although all isolates tested strongly positive by MHT and mCIM, Isolate B-1 demonstrated false susceptibility results to imipenem and meropenem by BMD (Table 4 and Figure 3). In addition, the two eCIM negative isolates showed much lower minimum inhibitory concentration (MIC) for cefepime, suggesting no or low level of *bla*_*cphA*7_ gene expression in these two isolates.

**Table 4:** Summary of phenotypic results of the three *Aeromonas hydrophila* isolates.

**Antibiotic class**	**Antibiotic**	***Aeromonas hydrophila*** **(11/20/18) A-1**		***Aeromonas hydrophila*** **(9/23/19) B-1**		***Aeromonas hydrophila*** **(10/10/19) B-2**	
		**MIC (MCG/mL)**	**Interpretation**	**MIC (MCG/mL)**	**Interpretation**	**MIC (MCG/mL)**	**Interpretation**
Beta-Lactams	Piperacillin/Tazobactam	>128	Resistant	>128	Resistant	>128	Resistant
	Ceftriaxone	>32	Resistant	>64	Resistant	>64	Resistant
	Ceftazidime	32	Resistant	>32	Resistant	>32	Resistant
	Ceftolozane/Tazobactam	16	No Inter Criteria	8	No Inter Criteria	16	No Inter Criteria
	Cefepime	8	Susceptible	4	Intermediate	>32	Resistant
	Imipenem	8	Resistant	1	Susceptible	>16	Resistant
	Meropenem	4	Resistant	1	Susceptible	>16	Resistant
	Ertapenem	>4	Resistant	>4	Resistant	>4	Resistant
	Ceftazidime/Avibactam	< =2	No Inter Criteria	< =2	No Inter Criteria	4	No Inter Criteria
Aminoglycoside	Gentamicin	1	Susceptible	< =1	Susceptible	>16	Resistant
	Tobramycin	8	No Inter Criteria	4	No Inter Criteria	>16	No Inter Criteria
	Amikacin	2	Susceptible	16	Susceptible	32	Intermediate
Trimethoprim-Sulfonamide	Trimethoprim-Sulfonamide	< =1/20	Susceptible	>4/80	Resistant	>4/80	Resistant
Colistin	Colistin	>4	No Inter Criteria	< =2	No Inter Criteria	>4	No Inter Criteria
Fluoroquinolone	Ciprofloxacin	>2	Resistant	>4	Resistant	>4	Resistant
	Levofloxacin	4	Intermediate	4	Intermediate	2	Susceptible
Modified Carbapenem Inactivation Method (mCIM)		Positive		Positive		Positive	
EDTA Carbapenem Inactivation Method (eCIM)		Negative		Negative		Positive	
Modified Hodge Test (MHT)		Positive		Positive		Positive	

## Discussion

Here we report the first cases of CphA-mediated carbapenem resistant *A. hydrophila* in the United States. The majority of CphA-mediated carbapenem resistance has been detected in soil or water environments in Asia and Europe (Walsh et al., 1997; Balsalobre et al., 2009; Piotrowska et al., 2017; Piccirilli et al., 2019), but also found in human infections as severe as bacteremia reported from Taiwan (Wu et al., 2007, 2011, 2012), Australia (Sinclair et al., 2016), and Colombia (Rosso et al., 2019). The idea that MDR organisms from the environment cause human infection is not novel; however, what is highlighted in this case is that current phenotypic methods are limited in correctly detecting carbapenem resistance in *Aeromonas* species. We demonstrated that using WGS and phenotypic carbapenemase assays can detect wider spectrum of resistance mechanisms that may be missed otherwise in highly resistant strains.

It is concerning that 1 out of 3 *A. hydrophila* isolates tested falsely susceptible to imipenem and meropenem by the conventional BMD method despite clear carbapenemase production shown by strongly positive MHT and mCIM results. These false susceptible results are defined by CLSI as very major errors (VMEs) (Humphries et al., 2018). VMEs are serious because they mislead clinicians to falsely believe that an ineffective antibiotic therapy is appropriate to administer. Current guidelines recommend ciprofloxacin or levofloxacin as first-line therapies for the infection with *Aeromonas* (Gilbert et al., 2019). Carbapenems are usually used empirically in treating Gram-negative bacteremia, as demonstrated in our cases. The carbapenem treatment in Patient B may have resulted in the selection of a sub-population of *A. hydrophila* that had an additional plasmid with antimicrobial resistance genes, or an induction of the carbapenemase gene CphA, which led to extended resistance profile (Table 3).

This work demonstrated the strength of using both mCIM and MHT phenotypic tests to detect the CphA-type carbapenemases in *A. hydrophila*. Currently the CLSI guidelines for the interpretation of mCIM results are limited in *Enterobacterales* and *Pseudomonas aeruginosa* while eCIM results are limited to only *Enterobacterales* (Pierce et al., 2017; Clinical Laboratory Standards Institute, 2019; Sfeir et al., 2019). MHT results are no longer endorsed by CLSI to be used for any species. Our study suggests that these phenotypic tests are useful for the detection of carbapenemase in *A. hydrophila*. The intriguing part is that we do not have an explanation for why the B-2 isolate tested positive for eCIM only after meropenem treatment. One possible explanation is that positive eCIM requires hyperproduced CphA enzyme, but MHT and mCIM do not. Further studies are needed to solve this puzzle.

The detection of chromosomally encoded β-lactamase genes in our study is consistent with previously published literature on *Aeromonas* species isolated from human infections (Janda and Abbott, 2010). However, the two Patient B isolates demonstrated a substantial increase in the number of antimicrobial genes detected genotypically with AMR prediction (Table 2). This increase in the number of antimicrobial genes detected genotypically is attributed to the presence of plasmids with one novel plasmid in both isolates and an additional plasmid in B-2 (Table 3). The ability of *Aeromonas* to exchange and gain plasmids is well-known within the literature and all species within *Aeromonas* are known to have an open pan-genome with extensive genomic variability (Bello-López et al., 2019; Zhong et al., 2019). Therefore, there is a greater ability for *Aeromonas* spp. to acquire AMR genes. Of note, in a recent pan-genome analysis, *A. aquatica* MX16A which was just recently re-classified as *A. hydrophila* MX16A (NZ_CP018201), was shown to harbor the greatest number of AMR genes among 29 species of *Aeromonas*. All three *A. hydrophila* isolates in our study were closely related to this strain. There was no known travel history or exposure history for these two patients. The epidemiological investigation revealed no common source or mode of transmission for the multidrug resistant *Aeromonas* strains isolated from these two patients. In addition, no other multidrug resistant *Aeromonas* were isolated or identified from sources or patients in the time period between the two patients. As of writing this manuscript, the sources of these *Aeromonas* were still unknown.

One major limitation of this study is that we did not perform further investigation to check if the *bla*_*cphA*7_ gene was derepressed in Patient B's Isolate B-2, which could provide more clear explanation for its much higher MICs for the carbapenems and cefepime compared to Isolate B-1. We did, however, identify a non-synonymous point mutation (AC) resulting in an Asp17Tyr substitution in the beta-lactam response regulator transporter gene *blrA* in both Isolate B1 and B2 (Supplementary Table 1). The gene *blrA* had been shown to regulate three inducible beta-lactamases encoded by *bla*_*ampH*_, *bla*_*cepH*_, and *bla*_*imiH*_ in an *A. hydrophila* strain T429125 (Niumsup et al., 2003). Further studies are required to elucidate how this mutation in the *blrA* gene affect the gene expression of various beta-lactamases in the isolates from Patient B.

Clinicians and clinical microbiologists in the US should be aware of the emerging MDR *Aeromonas* infections and the potential false carbapenem susceptible results due to CphA-type carbapenemase. The inability to accurately detect this type of carbapenemase using the conventional methods is concerning because carbapenem is a common drug choice for treating MDRO. Workflows within the clinical microbiology lab will need to adapt to detecting these type of resistance mechanisms in the future. We suggest one good strategy is combining both genomic analysis by WGS and phenotypic characterization by MHT, mCIM, and eCIM. Genomic analysis by WGS is not yet accessible for all clinical laboratories; therefore, we propose further studies be performed to establish a reliable and fast algorithm to detect these types of resistance mechanisms in *Aeromonas*. In addition, we suggest the susceptible carbapenem MIC results on *Aeromonas* species isolated from the sterile site in patients with severe infections should always be carefully evaluated before being finalized and reported.

## Data Availability Statement

The datasets generated for this study can be found in online repositories. The names of the repository/repositories and accession number(s) can be found in the article/Supplementary Material.

## Ethics Statement

Ethical review and approval was not required for the study on human participants in accordance with the local legislation and institutional requirements. Written informed consent for participation was not required for this study in accordance with the national legislation and the institutional requirements.

## Author Contributions

EH conceived of the presented idea, performed the experiments, analyzed the data, and drafted the manuscript. SF performed the experiments and analyzed the data. AM reviewed clinical history, and reviewed and revised the manuscript. KW performed the experiments. OG supervised the project, and reviewed and revised the manuscript. SY conceived of the presented idea, supervised the project, analyzed the data, and reviewed and revised the manuscript. All authors contributed to the article and approved the submitted version.

## Conflict of Interest

The authors declare that the research was conducted in the absence of any commercial or financial relationships that could be construed as a potential conflict of interest.
